# Pesticide exposure among Bolivian farmers: associations between worker protection and exposure biomarkers

**DOI:** 10.1038/s41370-019-0128-3

**Published:** 2019-02-20

**Authors:** Jessika Barrón Cuenca, Noemi Tirado, Max Vikström, Christian H. Lindh, Ulla Stenius, Karin Leander, Marika Berglund, Kristian Dreij

**Affiliations:** 1 Institute of Environmental Medicine, Karolinska Institutet, Box 210, SE-171 77 Stockholm, Sweden; 2 Genetic Institute, Medicine Faculty, Mayor of San Andres University, Saavedra Av., #2246 Miraflores, La Paz Bolivia; 3 Division of Occupational and Environmental Medicine, Lund University, Lund, 22363 Lund, Sweden

## Abstract

The use of pesticides has increased during the past decades, also increasing the risk of exposure to toxic pesticides that can cause detrimental health effects in the future. This is of special concern among farmers in low-to-middle-income countries that may lack proper training in the safe use of these chemicals. To assess the situation in Bolivia a cross-sectional study in three agricultural communities was performed (*n* = 297). Handling, use of personal protective equipment (PPE) and pesticide exposure were assessed by a questionnaire and measurements of urinary pesticide metabolites (UPMs). Results showed that methamidophos (65%) and paraquat (52%) were the most commonly used pesticides and that 75% of the farmers combined several pesticides while spraying. Notably, only 17% of the farmers used recommended PPEs while 84% reported to have experienced symptoms of acute pesticide poisoning after spraying. UPM measurements indicated high levels of exposure to chlorpyrifos, pyrethroids and 2,4D and that men generally were more highly exposed compared to women. Our study demonstrates that farmers who are better at following recommendations for pesticide handling and use of PPE had a significantly lower risk of having high UPM levels of most measured pesticides. Our results thus confirm the need of proper training of farmers in low-to-middle-income countries in proper protection and pesticide handling in order to reduce exposure levels and health problems.

## Background

Since the beginning of the 20th century, pests, including insects, rodents, fungi or unwanted plants are controlled or killed by pesticides. Pesticides have been used to promote public health by killing vectors of disease, and in agriculture to protect the crops avoiding low productivity. Pesticides can be classified based on their function as insecticides, herbicides, rodenticides, fungicides, etc., and based on type of chemical e.g., organophosphates, organochlorines, S-triazines and pyrethroids. In addition, pesticides are classified based on their toxic and carcinogenic potency by the World Health Organization (WHO) and the International Agency for Research on Cancer (IARC) [1, 2].

The use of pesticides can affect human health [3]. Exposure to these chemicals occurs especially among farmers and during application procedures by different routes of exposure such as dermal contact or inhalation. In order to reduce the exposure, there are international guidelines concerning personal protective equipment (PPE) which recommend that farmers who spray pesticides should protect themselves by covering most of their bodies [4]. This can however be a challenge in tropical countries where wearing PPE in a warm and humid climate may cause discomfort, resulting in reduced usage of this first line of defense against exposure to pesticides. Low use of PPE together with inadequate knowledge and awareness about handling and storage of pesticides constitute important issues that might increase the risk of exposure to pesticides among farmers [5, 6]. Residents of rural areas also may be affected due to the proximity to farms or ingestion of water or food that contains pesticide residues.

Exposure to pesticides can produce acute toxic effects with low (e.g., headache), moderate (e.g., diarrhea) or high (e.g. pulmonary edema) severity or even fatal intoxication [7]. In addition, long-term exposure could result in chronic health effects such as neurological, particularly neurodevelopmental abnormalities in children, and endocrine disruption resulting in precocious puberty [8, 9]. Farmers usually use different formulations of pesticides and in complex mixtures, some of which have genotoxic effects which could cause different types of cancers, including leukemia, multiple myeloma, malignant lymphomas, brain and prostate cancer [10–12].

This study was performed in Bolivia, and like other low-to-middle-income countries, Bolivian farmers have increasingly been using pesticides since the end of the last century in order to get into a competitive international agricultural market [13]. As a result, farmers have been applying pesticides to their crops without proper training or supervision, most likely increasing their exposure to these hazardous chemicals [14]. According to information extracted from the Bolivian Agricultural Census of 2013, 46% of the agricultural production units used pesticides to control pest and diseases of crops especially in the tropical area [15]. However, because of smuggling and under-reporting, the usage of these chemicals is not completely controlled and/or registered by the Bolivian authorities and thus probably underestimated. As a consequence, very little is known about which the most used pesticides are and the associated prevalence of health effects in farmers and in the general population in Bolivia. The aim of this study was to characterize exposure to pesticides among Bolivian farmers, and to assess the impact of behavior, including pesticide usage, handling and use of PPE, on exposure.

## Methods

### Study areas

**Fig. 1:**
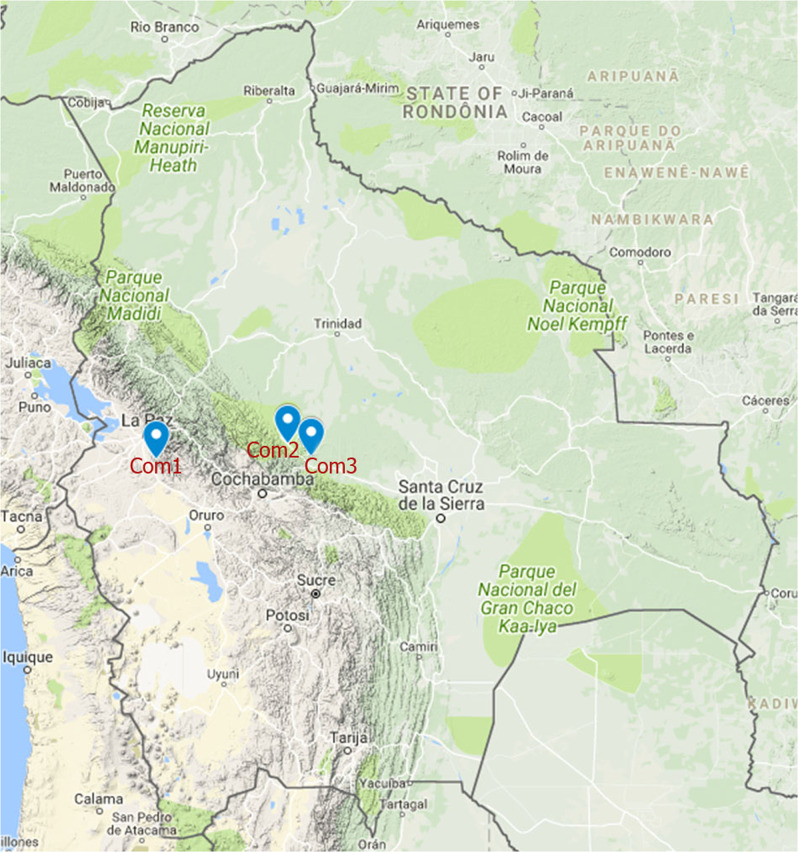
Map of Bolivia showing the three studied communities. Com1 (Sapahaqui in La Paz), Com2 and Com3 (respectively Villa Bolivar and Villa 14 de Septiembre in Cochabamba). Image used from Google maps (free online version) with modifications for the present study

This study was conducted in three different communities located in two different climate areas and altitudes in Bolivia; Sapahaqui (Com1) located in the Province of José Ramón Loayza, Department of La Paz; Villa Bolivar (Com2) and Villa 14 de Septiembre (Com3) located in Chapare-Cochabamba (Fig. 1). Farmers in these communities own small lands for cultivation, 1 600–15 000 m^2^ per family [15].

Com1 is situated at 3134  above sea level in a mountainous area. The climate is relatively warm throughout the whole year with a temperature around 13–20 °C, which makes this area ideal for the production of temperate crops such as tomatoes and celery. In Com1, ~79% of the population works as farmers and according to the census it had 12 484 inhabitants in 2012, of which almost all are of Aymara origin, natives of the Andes in Bolivia [16].

Com2 and Com3 belong to the tropical area of Bolivia, with a temperature between 25–30 °C, and at an altitude of ~200 meters above sea level. These conditions provide for more fertile fields and more harvests per year. Farms in this area produce high quantities of citrus, coca, and banana. In Com2 and Com3, around 65% of the populations are farmers. Com2 had 1 710 inhabitants and Com3 had 2 123 inhabitants according to the 2012 census [16]. These two communities have mainly a Quechua population, native people from the central parts of Bolivia.

### Recruitment and data collection

The study participants were selected with the following criteria: men and women who had lived in the communities for at least 5 years and age 17–70, because this age-group is actively involved in farming [15]. At the beginning of the study, we contacted local authorities and health promoters in each community in order to inform to the population about our project and the dates that our researchers would stay in the community. These promoters shared the information to the population orally and by radio advertisements. Since we do not know how many people this information reached, the response rate could not be estimated. At the day of sample collection, people were informed again by the team about the project and its aims, oral and written description of the project was given to the participants, and written informed consent was obtained prior to the interview. We estimate that up to 10% of the participants who showed up at the different locations left due to long waiting times. In total, 297 people participated in the study. Based on the gender and age distribution, and that we had participants from the two major climate zones of Bolivia, we consider that the study population is representative. The data were collected between June and October of 2015.

To characterize the exposure situation including lifestyle factors and handling of pesticides, trained members of our staff employed a short survey based on a questionnaire used in previous studies in Bolivia [14, 17]. The questionnaire (in Spanish and English) is provided as supplementary information. The interview form consisted of closed and open-ended questions including [1] population characteristics (e.g., age, community, gender, and education) [2], crops information (e.g. type of work, hours spent in the farm, and types of crops) [3], use of pesticides (e.g. type of pesticides or mixtures of pesticides, frequency of application, and handling) [4], information on personal protection equipment (PPE) (e.g. type of clothes for spraying, handling, storage and disposal of pesticide containers), and [5] health symptoms known to be related with acute pesticide poisoning (APP) consisting of the following five sections (a) nervous system, (b) respiratory system, (c) muscle system, (d) digestive system, and (e) epithelial and mucosal tissues.

All research procedures included in the study were conducted according to the principles of the Helsinki Declaration, and were reviewed and approved by the Ethical Committee for Research at Universidad Mayor de San Andrés in La Paz, Bolivia and the Regional Ethical Review Board in Stockholm, Sweden. All the personal data were pseudonymized and only the corresponding authors have access to the key to the identifiers.

### Urine sample collection and analysis

The day before the sample collection, our contacts in each community gave the volunteers an empty sterile polypropylene urine sample container in order to have the first morning urine collected the next day. Before starting the interview, each urine sample was collected and immediately aliquoted into a 10 mL polypropylene tube that was labeled and stored at –18 °C in a portable freezer (ARB, Alice Springs, Australia). The 297 samples were transported to the Genetic Institute in La Paz, Bolivia, and stored at –20 °C until further shipment on dry ice to Lund University (Sweden) for analysis. Urinary concentrations of ten pesticide metabolites (urinary pesticide metabolites, UPM) were analyzed; hydroxy-tebuconazole, a metabolite of tebuconazole (TEB-OH), 3,5,6-Trichloro-2-pyridinol, metabolite of chlorpyrifos (TCP), 3-phenoxybenzoic acid (3PBA) and sum of cis/trans 3-(2,2-dichlorovinyl)−2,2-dimethylcyclopropane carboxylic acid (DCCA) metabolites of pyrethroid insecticides, 2,4-dichlorophenoxyacetic acid (2,4D) and 4-chloro-2-methylphenoxy acetic acid (MCPA), metabolites of phenoxy herbicides, chloro-3,3,3-trifluoro-1-propen-1-yl]−2,2-dimethylcyclopropanecarboxylic acid (CFCA) metabolite of bifenthrin, 4-fluoro-3-phenoxybenzoic acid (4F3PBA) metabolite of pyrethroid cyfluthrin, 5-hydroxy-tiabendazole (5-OH-TBZ) metabolite of thiabendazole and 3-hydroxy-pyrimetanil (OH-PYR) metabolite of pyrimethanil.

Shortly, the urine samples were de-conjugated using β-glucuronidase/arylsulphatase and the samples were prepared using solid phase extraction. Quantitative analysis was conducted using a liquid chromatography-triple quadrupole linear ion trap mass spectrometer, (LC-MS/MS; QTRAP 5500; AB Sciex, Foster City, CA, USA) according to a modified method [18]. Limits of detection (LOD) for measuring TEB-OH, TCP, 3PBA, DCCA, 2,4D, MCPA, CFCA, 4F3PBA and OH-PYR were 0.10 ng/mL and for 5-OH-TBZ 0.05 ng/mL. The laboratory is part of an inter-laboratory control program for TCP and 3-PBA. Urinary density (g/mL) was also measured and used for normalization of concentrations. For samples with metabolite concentrations < LOD we used LOD/2.

### Statistical analysis

A data base in Excel 2010 was created where an individual code was assigned for each participant. All the questions were classified, codified and transferred to the Statistical Package for the Social Sciences software (SPSS Statistics 22). Pair wise statistical analysis of data from questionnaires and UPM measurements was performed by chi-square (categorical data, frequencies) or Student’s *T* test (continuous data). Multiple comparisons were performed by one-way ANOVA with multiple testing adjusted for by Bonferroni testing. In all tests, *p* < 0.05 was considered as statistical significant.

In order to analyze if the type of PPE used and how pesticides were handled were associated with risk of exposure to pesticides, we classified the participants based on use of PPE and handling of pesticides. Using the recommendations from the Food and Agriculture Organization of the United Nations (FAO) [4], a “protection and handling index” (PHI) score was created for each individual. Each PPE and how pesticides were handled were assigned a numerical value (SI Table 1) and the individual PHI score was calculated by summing the values where 16 points represented maximum protection and best behavior. Individuals with PHI score above median (median = 4 for all farmers) were regarded as following instructions satisfactory. Individuals with a UPM concentration above the 75th percentile were regarded as being highly exposed. Statistical analysis was performed by logistic regression with SAS 9.2. Since gender, age, BMI, source of drinking water, and geographical region could be confounders these were adjusted for. Confounding factors were selected based on expert knowledge.

## Results

### Population characteristics

A total of 297 people participated in the present study, consisting of 130 women (44%) and 167 men (56%) (Table 1). The average age was 42.2 years, with no major difference between genders. The educational level of the study population was low, 12% never went to the school and 62% went only to primary school. After weighing and measuring height of the participants and following the global database on body mass index (BMI) [19], we found that a larger proportion of women were obese (BMI ≥ 30) compared to the men (*p* < 0.001). Alcohol and tobacco consumption was more common among men (*p* < 0.001 both), but there were no heavy smokers or drinkers (Table 1). The majority of the study population had access to municipal water (37%) or a local well (34%). Other sources for drinking water were river, spring or rain water.

**Table 1:** Characteristics of the study population

Parameter	Total population	Com1	Com2	Com3
**Participants**	297	89 (30%)	107 (36%)	101 (34%)
**Age (in years)**				
Mean ± SD	42.2 ± 13.6	46.6 ± 15.5	38.5 ± 12.0	42.1 ± 12.3
**Gender (%)**				
Women	44	44	48	40
Men	56	56	52	60
**BMI**^a^ **(%)**				
Normal				
Women	26	41	26	12
Men	40**	46	43	33
Overweight				
Women	41	41	39	43
Men	51**	52	46	54
Obesity				
Women	33***	18**	35***	45***
Men	9	2	11	13
**Smoking habit**				
Yes (%)				
Women	13	3	22	12
Men	43***	32***	55***	41***
Cig/month ± SD				
Women	3.8 ± 4.1	2.0 ± 0.0**	3.7 ± 4.1	4.2 ± 4.9
Men	7.6 ± 11.8***	1.3 ± 1.0	4.8 ± 7.1*	15.2 ± 15.9**
**Alcohol consumption**				
Yes (%)				
Women	30	28	39	20
Men	49***	58*	75***	18
Units/month ± SD^b^				
Women	1.1 ± 0.3	1.0 ± 0.0	1.2 ± 0.5	1.0 ± 0.0
Men	1.4 ± 0.7***	1.3 ± 0.6**	1.4 ± 0.6***	1.7 ± 0.9
**Type of work (%)**				
Farmer				
Women	84	92	92	65
Men	99.5**	100	100	98
Non-farmer				
Women	16***	8*	8*	35***
Men	0.5	0	0	2
**Years being farmer (%)**				
<8 years				
Women	23	28	37	19
Men	17	16	23	13
>8 years				
Women	77	72	79	81
Men	83**	84	77	87

*p < 0.05; **p < 0.01, ***p < 0.001 by chi-square (for pair wise testing of total population) or one-way Anova with Bonferroni adjustment (for multiple testing including the 3 communities) and indicates higher frequency compared to the other gender

^a^Body mass index was determined as described [18]

^b^Alcohol consumption as units/month was determined as described [19]

A large majority of the participants were farmers (94%) and had been active for more than eight years. Among the non-farmers, women were more represented (*p* < 0.001, Table 1). No major differences in characteristics were observed between the three communities. The communities did however show a wide variation in the type of crops that were cultivated including different kinds of vegetables, fruits and cereals (SI Table 2). Coca leaves was the most cultivated crop in Com2 and Com3 (92 and 89%, respectively). In Com1, vegetables were more common with tomato (71%), celery (66%) and corn (62%) as the most common crops.

### Usage and handling of pesticides

All farmers (*n* = 275) reported use of pesticides with organophosphates, bipyridyl and pyrethroids being the most common. Some differences in which type of pesticides were most frequently used in the different communities were observed. Farmers in the more tropical Com2 and Com3 used organophosphates to a higher degree (97 and 92%, respectively) compared to farmers in Com1 (63%). In contrast, pyrethroids were more commonly used among farmers in Com1 (33%) compared to the other two communities (24 and 16%, respectively). Moreover, 30% of the farmers in Com1 reported to use sulfur as a fungicide, which farmers in Com2 and Com3 did not use at all.

The most commonly used pesticides were methamidophos (65%) followed by paraquat (52%) and glyphosate (43%) (Table 2). The first two are classified by WHO as belonging to class Ib and II, considered as highly and moderately hazardous, respectively [1]. Comparisons of the three communities showed some differences in their pesticide use. Farmers in Com1 did not use paraquat or glyphosate at all, but applied chlorpyrifos and profenofos, two organophophates that belong to class II, more frequently than the other two communities (*p* < 0.001). It was more common to use the carbamate methomyl (class Ib) among farmers in Com3 compared to the other communities (*p* < 0.01). Use of class Ib pesticides was however most common among farmers in Com2 (*p* < 0.01) with methamidophos being the most commonly used pesticide compared to the other communities (*p* < 0.001). The vast majority (96%) of the farmers reported the use of more than one pesticide and up to 13 pesticides were used by the same farmer (data not showed). It was also clear that pesticides were used as mixtures, 75% of the farmers mixed at least two pesticides for spraying their crops (Table 2).

**Table 2:** Frequency and behavior of pesticide use among farmers

Frequency (%) of most used pesticides^a^	Total farmers	Com1	Com2	Com3
Methamidophos (Organophosphate)	65	28	91***	70
Paraquat (Dipiridyl)	52	0	71	81
Glyphosate (OP—Phosphonate)	43	0	57	67
Cypermethrin (Pyretroid)	16	23	17	6
Imidacloprid (Neonicotinoid)	14	1	18	21
Mancozeb (Carbamate)	14	16	15	10
Chlorpyrifos (Organophosphate)	13	27***	6	7
Methomyl (Carbamate)	12	1	11	24***
Lambdacyhalothrin (Pyretroid)	10	9	12	10
Profenofos (Organophosphate)	10	30***	2	0
**Frequency (%) according to toxicological classification**^b^				
Ia (Extremely hazardous)	0	0	0	0
Ib (Highly hazardous)	28	19	36**	27
II (Moderately hazardous)	35	37	32	38
III (Slightly hazardous)	4	6	3	4
II—III (In between)	2	6	0	0
U (Unlikely to present acute hazard)	31	32	29	31
**Use of pesticide mixtures**^c^ (%)				
Do not remember	13	26	1	13
Do not mix	12	5	10	22
Mix pesticides	75	69	89	65
**Days spraying per month (%)**				
1 day	9	8	8	12
2–10 days	38	49**	40	26
11–20 days	15	30***	12	3
More than 20 days	38	13	40***	59**
*Information source (%)*				
Read label instructions	16	37***	8	4
Agricultural engineer	10	13	16	0
Own experience	17	22	20	8
From the seller of pesticides store	57	28	56	88
**Amount of pesticide used for spraying (%)**				
Do not remember	2	2	2	1
Do not measure	26	29	28	22
Recommended amount	72	69	70	77
**Chewing coca while spraying**				
Yes (%)	72	63	81*	70

*p  ≤ 0.05; **p ≤ 0.01, ***p ≤ 0.001 by one-way Anova with Bonferroni adjustment and indicates higher frequency compared to the other community/ies

^a^Total information over 275 farmers divided by community; Com1 = 86 farmers, Com2 = 103 farmers, Com3 = 86 farmers. Frequency shows the ten most common pesticides by name and family

^b^Classification according to WHO [20]

^c^Mixture of pesticides: more than one pesticide used for the same crop and sprayed at the same time

The spraying frequency was in general higher in the two tropical communities with > 20 days of spraying per month compared to Com1 where most farmers sprayed 2–10 days per month (*p* < 0.01). Similarly, men spent significantly more days per month spraying than women (*p* < 0.01) although the same amount of hours per day working on the farm were reported for men and women. The most common source of information about how much pesticide to apply was information obtained from the retailer (57%, Table 2). In Com1, a larger proportion of the farmers also relied on information found on the label of the pesticide container (37% *p* < 0.001). Notably, 26% of the farmers stated that they did not measure the amount of pesticide they used. In agreement with the strong cultural habit of chewing coca leaves in Bolivia, 72% of all farmers stated that they chewed coca while spraying pesticides, something that was more common among farmers in Com2 (*p* < 0.05, Table 2).

### Use of personal protection equipment (PPE)

Only 41% stated that they used at least one piece of clothing as personal protection equipment (PPE). This number was lower in Com1 than in the other two communities (Table 3). Notably, only 17% of all the farmers were well protected according to recommendations from FAO. In all communities, fewer women were well protected compared to men (*p* < 0.01, Table 3).

**Table 3:** PPE habits and pesticide handling among farmers (*n* = 275)

Use of PPE (%)	Total farmers	Com1	Com2	Com3
Using protection equipment	41	24	53	44
**Well protected**^a^				
Total	17	33	9	18
Women	4	0	3	11
Men	28**	47*	24*	21
**Type of PPE (%)**^**b**^				
Hat	76	57	84*	76
Mask/Scarf	25	29	25	21
Boots	20	0	14	39**
Gloves	10	14	9	8
Glasses	8	14	4	11
Overall	8	29***	2	5
Apron	5	14*	0	8
**Change clothes after spray pesticides (%)**				
Yes	73	71	77	72
**Place to store spraying clothes (%)**				
With all the clothes	64***	40	74	77
Outside house^c^	36	60***	26	23
**Place to store pesticides and equipment (%)**				
Do not remember	2	0	2	1
Inside house	39	9	42	55
Outside house^c^	59	91***	56	44
**Disposal method of empty pesticides containers (%)**				
Do not know	3	5	4	1
Burning	38	17	42	56
Store them/Trash	31	49*	21	24
Throw them in the local river	27	29	33	19

*p ≤ 0.05; **p ≤ 0.01, ***p ≤ 0.001 by chi-square (for pair wise testing of total population) or one-way Anova with Bonferroni adjustment (for multiple testing including the 3 communities)and indicates higher frequency compared to the other gender or community/ies.

^a^According to FAO Guidelines the minimum requirement for all types of pesticide operations is lightweight clothing covering most of the body. Farmers were deemed as well protected when using an overall alone or with any other clothing covering parts of the body (hat, boots, mask/scarf, gloves, glasses or apron) or at least three of these items [4].

^b^Frequencies represent the 114 farmers stating that they used at least one PPE.

^c^Outside house includes: backyard, barnyard or shed

The by far most common PPE in all communities was hat (76%). A higher proportion of farmers in Com1 used an overall or apron (*p* < 0.001 and *p* < 0.05, respectively) while boots where most common in Com3 (*p* < 0.01). Most of the farmers stated that they changed clothes after spraying, with no significant differences between the habits of women and men or between communities. When it came to storage of PPE, pesticides and related equipment, farmers in Com1 to a much higher degree stored them outside the house (Table 3). Regarding pesticide disposal, most farmers in Com2 and Com3 used to burn their empty pesticide containers while it was more common to store or trash them in Com1. Notably, 27% of all farmers stated that they throw empty containers in the local river. This is especially worrying since 61% of all farmers also stated the river as their main source of water for watering the crops or their own consumption.

### Health effects related with exposure to pesticides

The crops in these areas grow close to the farmer’s houses, and as a result, 75% of the farmers reported to have felt pesticide odor around their living area. When asked whether the farmers had experienced signs or symptoms of acute health effects during or after spraying with pesticides, 80% of the farmers reported to have experienced symptoms at least once, and women more frequently than men (*p* < 0.05, Table 4). 52% of the farmers reported to have had more than three different symptoms at the same time which can be catalogued as acute pesticide poisoning (APP) [7, 20, 21]. Headache was the most common symptom and especially among women in Com1 and Com2 (*p* < 0.05 in both). Burning eyes, dizziness and red skin were also among the most common symptoms. More men than women had experienced dizziness (*p* < 0.05) but more women reported to have experienced shaking chills (*p* < 0.01). Among the farmers in Com2, headaches, burning eyes, red skin and shaking chills were all significantly more common among women (*p* < 0.05–0.001, Table 4) compared to men and in agreement with the large differences in use of PPE among men and women in Com2.

**Table 4:** Acute health symptoms experienced by farmers during and/or after spraying pesticides

Parameter	Total farmers	Com1	Com2	Com3
**Ever felt sick after spraying pesticides (%)**				
Yes				
Total	80	67	88	84
Women	84*	72	89	92
Men	78	64	87	80
**The most common signs and symptoms**^a^ **(%)**				
*Nervous system*				
Headache				
Women	80	81*	88*	67
Men	70	50	67	85
Dizziness				
Women	29	35	31	21
Men	46*	41	53*	42*
Fatigue				
Women	16	19	14	17
Men	15	12	20	10
*Respiratory system*				
Dyspnea				
Women	11	15	12	4
Men	8	16	6	4
Cough				
Women	11	15	12	4
Men	6	16	6	0
*Muscular system*				
Cramp				
Women	14	15	17	8
Men	9	9	6	12
Fasciculation				
Women	17**	19	19*	13
Men	5	6	4	6
*Digestive system*				
Abdominal pain				
Women	31	15	36	42
Men	32	12	41	35
Nausea				
Women	29	19	33	33
Men	22	9	26	25
Vomiting				
Women	29	11	45	21
Men	23	16	33	19
Red skin				
Women	41	35	57***	21
Men	36	28	29	50*
Itchy skin				
Women	17	39	14	0
Men	14	37	8	4
Eyes burning				
Women	52	35	67***	46
Men	42	62*	24	46
Red eyes				
Women	13	15	17	4
Men	18	37	14	8

*p < 0.05; **p < 0.01, ***p < 0.001 by chi-square (for pair wise testing of total population) or one-way Anova with Bonferroni adjustment (for multiple testing including the 3 communities) and indicates higher frequency compared to the other gender or community/ies

^a^Data over 221 farmers (129 men and 92 women) who stated that they have had at least one sign or symptom of acute intoxication by pesticides

About a third of all female farmers reported having sprayed pesticides meanwhile they were pregnant or breast feeding, something that was most common in Com2 with 56 and 44%, respectively (SI Table 3). Almost half of all the women reported to have had miscarriages and 17% having delivered a child with a malformation/still birth, with no significant differences between communities or between women being farmers or not.

### Urinary concentrations of pesticide metabolites

The two pyrethroid metabolites 3PBA and DCCA and the organophosphate metabolite TCP was detected in all urine samples. For the other metabolites, the frequency of detection ranged between 2 and 93%. The UPM concentrations among the study population are shown in Table 5. Highest mean concentrations were detected for TCP (17.6 ng/ml) and 2,4D (15.8 ng/ml). Maximum concentrations were for some biomarkers more than 100-fold higher than their mean concentrations.

**Table 5:** Urinary concentrations of pesticide metabolites in the study population (ng/ml)

Pesticide(s)	UPM	Detection frequency (%)		Min	Mean	Max	IQR
Tebuconazole	TEB-OH^a^	93	Total	<LOD	3.18	458	0.243–1.42
			Women	<LOD	1.38	23.5	0.231–0.945
			Men	<LOD	4.59	458	0.263–1.70
Chlorpyrifos	TCP	100	Total	0.779	17.6	439	3.09–12.2
			Women	0.779	17.2	413	2.82–11.1
			Men	0.856	17.9	439	3.40–12.9
Permethrin, cypermethrin, and cyfluthrin	3PBA	100	Total	0.156	3.22	40.3	0.988–3.36
			Women	0.156	2.44	15.2	1.01–2.95
			Men	0.189	3.81***	40.3	0.962–3.95
	DCCA	100	Total	0.141	5.02	156	1.14–5.31
			Women	0.271	4.17	15.2	1.02–4.78
			Men	0.141	5.68	156	1.21–5.32
Phenoxy herbicides	2,4D	89	Total	<LOD	15.8	1 705	0.167–0.804
			Women	<LOD	1.51	33.9	0.148–0.659
			Men	<LOD	26.9**	1 705	0.196–0.964
	MCPA	2	Total	<LOD	0.0541	0.392	<LOD
			Women	<LOD	0.0528	0.358	<LOD
			Men	<LOD	0.0552	0.392	<LOD
Cyfluthrin and bifenthrin	CFCA	74	Total	<LOD	0.365	11.4	<LOD − 0.341
			Women	<LOD	0.296	5.58	<LOD − 0.283
			Men	<LOD	0.418	11.4	<LOD − 0.370
	4F3PBA	12	Total	<LOD	0.147	3.94	<LOD
			Women	<LOD	0.192**	3.94	<LOD
			Men	<LOD	0.113	2.20	<LOD
Thiabendazole and pyrimethanil	5-OH-TBZ	5	Total	<LOD	0.0534	4.10	<LOD
			Women	<LOD	0.0803**	4.10	<LOD
			Men	<LOD	0.0324	1.00	<LOD
	OH-PYR	10	Total	<LOD	2.41	395	<LOD
			Women	<LOD	0.762	54.0	<LOD
			Men	<LOD	3.70*	395	<LOD

*p < 0.05, **p < 0.01, ***p < 0.001 by Student’s T-test and indicates higher UPM levels compared to the other gender

^a^For abbreviations, see materials and methods

**Fig. 2:**
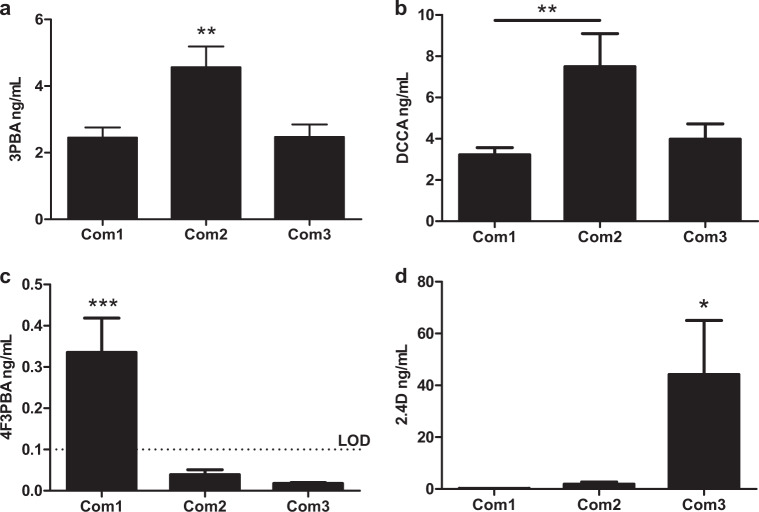
Differences in UPM concentrations between the 3 communities. Significantly different concentrations of 3PBA (**a**), DCCA (**b**), 4F3PBA (**c**) and 2,4D (**d**) were found between the 3 communities. LOD = limit of detection. *p < 0.05; **p < 0.01; ***p < 0.001 by one-way ANOVA with Bonferroni adjustment

Comparing concentrations found in urine from men and women, the metabolites 3PBA, 2,4D and OH-PYR were found at significantly higher concentrations among men (*p* < 0.05–0.001) and 4F3PBA and 5-OH-TBZ, were found at significantly higher concentrations among women (*p* < 0.01). Comparing across communities (Fig. 2), it was found that participants in Com2 had significantly higher concentration of the two pyrethroid metabolites 3PBA and DCCA in urine than the other two communities (*p* < 0.01 for both). Participants in Com1 showed significantly higher urine concentrations of the pyrethroid metabolite 4F3PBA than the other communities (*p* < 0.001). Similarly, highest concentrations of 2,4D were found in Com3 (*p* < 0.001), which is in agreement with 2,4D reported to almost exclusively being used in this community (SI Table 4).

### Relationships between lifestyle, PPE, pesticide handling and urine pesticide concentrations

**Fig. 3:**
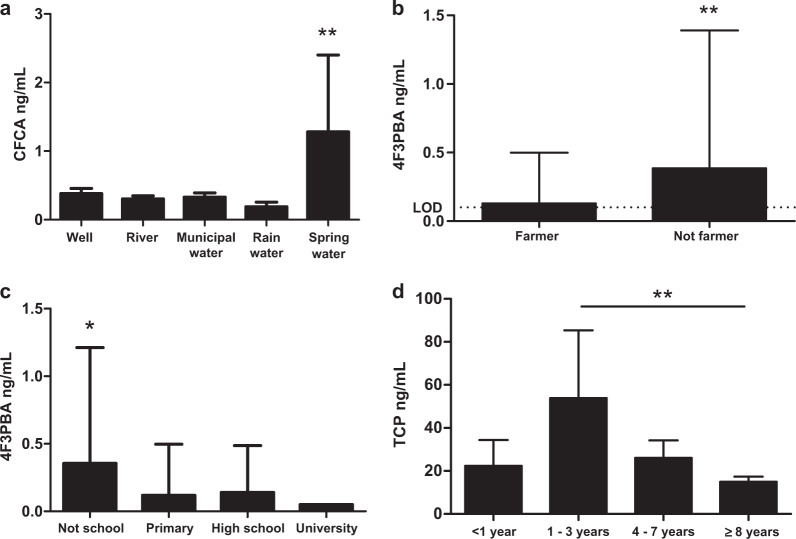
Impact of life style factors on UPM concentrations. UPM concentrations of CFCA, 4F3PBA and TCP were significantly affected by source of drinking water (**a**), type of occupation (**b**), level of education (**c**) and years working as farmer (**d**), respectively. *p < 0.05; **p < 0.01 by Student T-test or one-way ANOVA with Bonferroni adjustment

Highest concentrations of CFCA were found in people who used spring water as source of drinking water in comparison with those who consumed water from other sources (*p* < 0.05, Fig. 3a). 4F3PBA showed higher concentrations in the participants not actively working as farmers (*p* < 0.01, Fig. 3b), suggesting a different source of exposure than farming. The same metabolite was found at significantly higher concentrations in participants without education (not attending school at all) (*p* < 0.05, Fig. 3c). The period of time working as a farmer could be an important factor of exposure. No clear correlations between number of days spent spraying per month and UPM concentrations were observed. However, significantly higher concentrations of TCP among farmers working 1–3 years compared to those working more than 8 years (*p* < 0.05, Fig. 3d) was the only observed effect. Moreover, 3PBA concentrations showed high concentrations in people who disposed of empty pesticide containers in the river compared to other means of disposal (*p* < 0.05). The habit of chewing coca leaves while spraying with pesticides was not correlated with increased levels of any of the measured UPMs.

To investigate the impact of type of PPE used and handling of pesticides on the risk of having increased UPM concentrations a PHI score was calculated as described in materials and methods. Comparing the PHI scores between the three communities showed that Com1 had the highest median score (PHI = 5) followed by Com2 and Com3 (PHI = 4 and 3, respectively) which is in agreement with results shown in Tables 2 and 3. Regression analysis between PHI score and UPM concentrations indicated a reduced risk (OR < 1) for all metabolites except TCP, which instead showed an increased risk (Table 6). A significant protective effect was however only observed for the pyrethroid metabolites 3PBA and DCCA with ORs of 0.44 (95% CI: 0.24–0.81) and 0.52 (95% CI: 0.28–0.97), respectively. The same reduced risk for these two metabolites was observed when adjusting for gender and age. When also adjusting for geographical area (La Paz (Com1) and Cochabamba (Com2 and Com3)), water source and BMI, only the protective effect against 3PBA remained with OR = 0.50 (0.26–0.95).

**Table 6:** Impact of PHI score on risk of having high concentrations of UPM

UPM^a^	Crude OR	95% CI	*p*-value	adj. OR^b^	95% CI	*p*-value	adj. OR^c^	95% CI	*p*-value
TEB-OH	0.74	0.41–1.32	0.30	0.77	0.43–1.38	0.38	0.96	0.52–1.79	0.90
TCP	1.47	0.85–2.55	0.17	1.47	0.82–2.55	0.18	1.13	0.63–2.04	0.68
3PBA	0.44	0.24–0.81	0.009**	0.45	0.24–0.83	0.01*	0.50	0.26–0.95	0.03*
DCCA	0.52	0.28–0.97	0.03*	0.53	0.29–0.98	0.04*	0.57	0.30–1.07	0.07
2,4D	0.78	0.43–1.40	0.41	0.78	0.43–1.40	0.40	1.14	0.60–2.19	0.69
CFCA	0.62	0.34–1.12	0.11	0.62	0.34–1.14	0.12	0.71	0.38–1.32	0.27

*p < 0.05; **p < 0.01 by logistic regression and indicates a reduced risk of high UPM concentrations compared to farmers with low PHI score

^a^Metabolites MCPA, 4F3PBA, 5-OH-TBZ and OH-PYR were left out from the analysis due to low detection frequency (see Table 5)

^b^Model adjusted by gender and age

^c^Model adjusted by gender, age, geographical area, source of water and BMI

## Discussion

Although previous studies have indicated that farmers in Bolivia probably are exposed to high levels of pesticides, this is the first study measuring UPM concentrations representing a large number of different pesticides in a Bolivian population. Highest mean concentrations were obtained for TCP, a biomarker for exposure to chlorpyrifos, which was used in all communities, and for the herbicide 2,4D, which was used almost exclusively in Com3. Both of these metabolites have been measured in several studies, and the mean levels of TCP and 2,4D in our population are up to 50-fold higher than background levels in the general population of USA [22] but quite similar to levels found among farmers in Iowa, USA [23]. Women active as farmers in low-to-middle-income countries seem to be a vulnerable group [24, 25]. The Bolivian women in our study had up 40-fold higher levels of urinary TCP compared to women in Puerto Rico, Norway and Netherlands [26, 27]. Similarly, levels of 2,4D were about four times higher in our women compared to women from Puerto Rico [26]. Although our results show that women clearly are less protected while spraying, both mean and maximum UPM concentrations were higher among men for most pesticides. This can probably be explained by that fact that the men were much more active in spraying compared to women and thus more highly exposed. A limitation of the study is that the pesticides reported to be used by most farmers, the organophosphate methamidaphos and the herbicide paraquat, were not measured in the collected urine samples. Human biological monitoring studies have however shown that methamidaphos has a very short half-life making it challenging to be studied in occupational or environmental exposure settings [28]. The methodology for measuring a biomarker for paraquat was not available.

The importance of using recommended PPE and following recommendations for handling pesticides in limiting the exposure to pesticides was confirmed in this study. The results showed a correlation between following recommendation and decreased risk of high urinary concentrations for all UPMs except TCP. This was especially clear for the pyrethroid biomarkers and when adjusted for gender, age and geographical area. Notably, when adjusting for geographical area, the ORs for the other UPMs became closer to 1, indicating that there is some confounding factor related to area which we in this study were unable to further determine due to lack of power if we were to stratify the analysis based on area. The inverse relationship between good protection/behavior and urinary concentrations of TCP is difficult to interpret but might suggest another important source of exposure to chlorpyrifos such as contaminated food.

The consequences of the mishandling of pesticides and low use of PPE were also reflected in the large number of farmers that reported to have experienced APP after spraying pesticides. In agreement with previous occupational field studies, the poisonings were not severe but rather minor [29]. The wide variety of symptoms reported in our study population is probably a result of the combined effects of the many used pesticides. Notably, only 4% of the women wore the recommended PPE, which probably explains the high incidence of headaches, red skin and burning eyes. A possible explanation for the high number of symptoms could also be dehydration, long working days and, sun burn etc. As in many previous studies, a large proportion of the women reported to have sprayed pesticides while being pregnant or breastfeeding [30, 31]. A number of studies have found an association between pesticide exposure during pregnancy and increased risk of spontaneous miscarriages [24, 32]. In this study, a large proportion of the women (48%) reported having had a spontaneous miscarriage, a number that was similar among farmers and non-farmers. This is much higher than the data published by the Ministry of Health in Bolivia, which reported a frequency of respectively 14 and 22% in La Paz and Cochabamba in 2015 [33]. Similarly, a higher frequency of malformation and stillbirths (17%) was found in our study compared to previous reported levels (2.2%) [34]. These high frequencies might be due to a lack of statistical and/or non-objective information about these health effects from rural communities.

Since the three communities under study were mainly agricultural, the absolute majority of the participants were farmers and had been active as such for more than 8 years. In accordance with previous studies looking at handling and exposure to pesticides among agricultural communities in low-to-middle-income countries, the education level was low; 74 % never went to school or only to primary school [6, 35, 36]. In contrast to the Bolivian agricultural census of 2013 [15], which stated that 46% of the production units used pesticides, the results presented here indicate that the use of pesticides is much higher. All of the farmers used at least one pesticide, methamidophos being the most common and in agreement with a previous study from Com1 in Bolivia [14]. In agreement with a recent study from India [6], a majority of the 49 pesticides reported to be used are highly or moderately hazardous to human health according to the WHO classification [1]. It was moreover clear that the majority of the farmers used a mixture of pesticides when spraying their crops. This is most likely in order to control a variety of pests for the different crops that are being grown and in agreement with other studies assessing the use of pesticides among farmers in low-to-middle-income countries [37, 38].

Some farmers in Com1 have previously been part of an Integrated Pest Management (IPM) project run by the non-governmental organization PLAGBOL (Plaguicidas Bolivia) between 2001 and 2010 where they were trained in alternative ways to control pest and in the safe handling of pesticides [39–41]. Information about which farmers had previously been trained through this project was not available to us. Farmers of the other two communities have had no previous training on these issues by this or any other organization. Although previous training on the use of pesticides and their health effects on humans has taken place within the community, farmers in Com1 participating in this study appears to continue using highly or moderately toxic pesticides, although to a lesser extent than the two other communities. This might be explained by the fact that replacing pesticides with less hazardous compounds and IPM are both processes which take time. In addition, and although this is in contrast to what has been reported from the PLAGBOL project [41], the know-how might not have spread outside the families/farmers that were included in the project. The lower spraying frequency in Com1 compared to the two other communities is probably a result of differences in climate and type of crops being cultured, but could also be due to increased awareness about IPM. A difference was however observed between Com1 and the two other communities regarding handling of pesticides and the use of PPE. Farmers in Com1 read and followed product labels on how to use pesticides, were well protected (PPE), and stored and disposed of equipment and pesticides according to recommendations to a significantly higher degree compared to the other two communities. This suggests that the Com1 farmers in our study, directly or indirectly, have received information or training about proper pesticide usage and handling.

Previous studies performed in Bolivia related with the training in handling of pesticides and impact on health effects were performed in small groups of farmers where most of them were men [14, 41]. Our study took a representative higher number of participants where almost half were women actively involved in farming and thus better representing the agricultural communities in Bolivia. In agreement with previous studies performed in Bolivia, we show that farmers are using a large variety of pesticides and that most farmers are not following recommendations for proper handling and protection. By biomarker measurements of urinary pesticide metabolites, we can for the first time show that the agricultural population in Bolivia is exposed to high levels of pesticides. Moreover, results from this study confirms the need and importance of education and training of farmers in low-to-middle-income countries in proper protection and handling of pesticides in order to reduce exposure levels and detrimental health effects.

### Limitations of the study

In general, cross-sectional studies are very useful for investigating prevalence of exposure-related diseases. However, the limitations of this kind of studies include information biases, and the fact that the risk factors and outcomes are measured at the same time, which is why causation has to be confirmed rigorously. A weakness of the study was also the lack of information about times sprayed last month, and time from last spray to urine sampling. The information given about spraying frequency per month should be considered to be more general. These limitations could possibly explain why no correlation was observed between spraying frequency and levels of UPM. Recall bias was a likely limitation when the participants were asked to list which pesticides that they were using. This could have resulted in an underestimation of which pesticides were used among the farmers. Similarly, a relatively high number of participants stated that they did not remember if they used mixtures of pesticides or not. In both cases women answered that they did not remember to a higher degree. Moreover, our UPM analyses did not include the two most used pesticides, metamidophos and paraquat, which limits the quantitative assessment of pesticide exposure among Bolivian farmers as well as the analysis of the protective effect of using PPE or correlation with health effects.

## Supplementary information

Supplementary Tables 1–4

## References

[1] IPCS. WHO recommended classification of pesticides by hazard and guidelines to classification 2009. Albany, Switzerland: World Health Organization; 2010.

[2] International agency for research on cancer. iarc monographs—classifications: international agency for research on cancer. 2017. http://www.monographs.iarc.fr/ENG/Classification/latest_classif.php. Accessed september 2018.

[3] Kim KH, Kabir E, Jahan SA. Exposure to pesticides and the associated human health effects. Sci Total Environ. 2017;575:525–35. 10.1016/j.scitotenv.2016.09.009 27614863

[4] Food and Agriculture Organization of the United Nations. Guidelines for personal protection when working with pesticides in tropical climates [Work protection]. Rome: 1990;1990. http://www.fao.org/fileadmin/templates/agphome/documents/Pests_Pesticides/Code/Old_guidelines/PROTECT.pdf. Accessed september 2018.

[5] Karunamoorthi K, Mohammed M, Wassie F (2012). Knowledge and practices of farmers with reference to pesticide management: implications on human health. Arch Environ Occup Health.

[6] Banerjee I, Tripathi SK, Roy AS, Sengupta P (2014). Pesticide use pattern among farmers in a rural district of West Bengal, India. J Nat Sci Biol Med.

[7] Thundiyil JG, Stober J, Besbelli N, Pronczuk J (2008). Acute pesticide poisoning: a proposed classification tool. Bull World Health Organ.

[8] Mostafalou S, Abdollahi M (2013). Pesticides and human chronic diseases: evidences, mechanisms, and perspectives. Toxicol Appl Pharmacol.

[9] Hansen MRH, Jors E, Lander F, Condarco G, Debes F, Bustillos NT (2017). Neurological deficits after long-term pyrethroid exposure. Environ Health Insights.

[10] Bolognesi C (2003). Genotoxicity of pesticides: a review of human biomonitoring studies. Mutat Res.

[11] Dich J, Zahm SH, Hanberg A, Adami HO (1997). Pesticides and cancer. Cancer Causes Control.

[12] Bassil KL, Vakil C, Sanborn M, Cole DC, Kaur JS, Kerr KJ (2007). Cancer health effects of pesticides: systematic review. Can Fam Physician.

[13] Sanchez-Guerra M, Perez-Herrera N, Quintanilla-Vega B (2011). Organophosphorous pesticides research in Mexico: epidemiological and experimental approaches. Toxicol Mech Methods.

[14] Jors E, Morant RC, Aguilar GC, Huici O, Lander F, Baelum J (2006). Occupational pesticide intoxications among farmers in Bolivia: a cross-sectional study. Environ Health.

[15] Bolivian National Institute of Statistics. Agricultural census of Bolivia 2013 La Paz, Bolivia. 2015. http://www.sudamericarural.org/images/en_papel/archivos/CENSO-AGROPECUARIO-BOLIVIA_final.pdf. Accessed september 2018.

[16] Estado Plurinacional Bolivia. Censo Poblacional y Vivienda 2012 La Paz—Bolivia: INE. 2015. http://www.datos.ine.gob.bo. Accessed september 2018.

[17] Jors E, Gonzales AR, Ascarrunz ME, Tirado N, Takahashi C, Lafuente E (2007). Genetic alterations in pesticide exposed Bolivian farmers: An evaluation by analysis of chromosomal aberrations and the comet assay. Biomark Insights.

[18] Ekman E, Faniband MH, Littorin M, Maxe M, Jonsson BA, Lindh CH (2014). Determination of 5-hydroxythiabendazole in human urine as a biomarker of exposure to thiabendazole using LC/MS/MS. J Chromatogr B Anal Technol Biomed Life Sci.

[19] WHO. World Health Organization 2017. http://www.apps.who.int/bmi/index.jsp?introPage=intro_3.html. Accessed september 2018.

[20] Calvert GM, Karnik J, Mehler L, Beckman J, Morrissey B, Sievert J (2008). Acute pesticide poisoning among agricultural workers in the United States, 1998–2005. Am J Ind Med.

[21] Roberts JR, Reigart JR. Recognition and management of pesticide poisonings: EPA, United States environmental protection agency. 2013. https://www.epa.gov/sites/production/files/2015-01/documents/rmpp_6thed_final_lowresopt.pdf. Accessed september 2018.

[22] CDC. Fourth National Report on human exposure to environmental chemicals. Atlanta, GA: Centers for Disease Control and Prevention. 2018. https://www.cdc.gov/exposurereport/pdf/fourthreport_updatedtables_volume1_mar2018.pdf. Accessed 2018-05-02.

[23] Brian DC, Misty JH, Wayne TS, Dana BB, Dick H, Stephen JR (2005). Urinary and hand wipe pesticide levels among farmers and nonfarmers in Iowa. J Expo Anal Environ Epidemiol.

[24] Naidoo S, London L, Burdorf A, Naidoo R, Kromhout H (2011). Spontaneous miscarriages and infant deaths among female farmers in rural South Africa. Scand J Work Environ Health.

[25] Murphy HH, Sanusi A, Dilts DR, Yuliantiningsih S, Djajadisastra M, Hirschhorn N (2000). Health Effects of Pesticide Use Among Indonesian Women Farmers. J Agromedicine.

[26] Lewis RC, Cantonwine DE, Anzalota Del Toro LV, Calafat AM, Valentin-Blasini L, Davis MD, et al. Urinary biomarkers of exposure to insecticides, herbicides, and one insect repellent among pregnant women in Puerto Rico. Environmental Health. 2014;13:97. 10.1186/1476-069X-13-97. 10.1186/1476-069X-13-97 PMC4258050 25409771

[27] Ye X, Pierik FH, Angerer J, Meltzer HM, Jaddoe VWV, Tiemeier H (2009). Levels of metabolites of organophosphate pesticides, phthalates, and bisphenol A in pooled urine specimens from pregnant women participating in the Norwegian Mother and Child Cohort Study (MoBa). Int J Hyg Environ Health.

[28] Garner F, Jones K (2014). Biological monitoring for exposure to methamidophos: a human oral dosing study. Toxicol Lett.

[29] Litchfield MH (2005). Estimates of acute pesticide poisoning in agricultural workers in less developed countries. Toxicol Rev.

[30] Mrema EJ, Ngowi AV, Kishinhi SS, Mamuya SH (2017). Pesticide exposure and health problems among female horticulture Workers in Tanzania. Environ Health Insights.

[31] Murphy HH, Sanusi A, Dilts R, Yuliatingsih S, Djajadisastra M, Hirschhorn N (2000). Health effects of pesticide use among indonesian women farmers. J Agromedicine.

[32] Petrelli G, Figa-Talamanca I, Tropeano R, Tangucci M, Cini C, Aquilani S (2000). Reproductive male-mediated risk: spontaneous abortion among wives of pesticide applicators. Eur J Epidemiol.

[33] Health Ministry of Bolivia. National Health Information System. Epidemiological surveillance (SNIS-VE). 2018. http://snis.minsalud.gob.bo/. Accessed september 2018.

[34] Nazer HJ, Cifuentes OL (2011). Malformaciones congénitas en Chile y Latino América: Una visión epidemiológica del ECLAMC del período 1995-2008. Rev médica De Chile.

[35] Shomar B, Al-Saad K, Nriagu J (2014). Mishandling and exposure of farm workers in Qatar to organophosphate pesticides. Environ Res.

[36] Jors E, Hay-Younes J, Condarco MA, Condarco G, Cervantes R, Huici O (2013). Is Gender a Risk Factor for Pesticide Intoxications Among Farmers in Bolivia? A Cross-Sectional Study. J Agromedicine.

[37] Ngowi AVF, Mbise TJ, Ijani ASM, London L, Ajayi OC (2007). Smallholder vegetable farmers in Northern Tanzania: pesticides use practices, perceptions, cost and health effects. Crop Prot.

[38] Houbraken M, Bauweraerts I, Fevery D, Van Labeke MC, Spanoghe P (2016). Pesticide knowledge and practice among horticultural workers in the Lam Dong region, Vietnam: A case study of chrysanthemum and strawberries. Sci Total Environ.

[39] Jors E, Lander F, Huici O, Cervantes Morant R, Gulis G, Konradsen F (2014). Do Bolivian small holder farmers improve and retain knowledge to reduce occupational pesticide poisonings after training on Integrated Pest Management?. Environ Health.

[40] PLAGBOL. Fundación Plagbol La Paz—Bolivia. 2017. http://www.plagbol.org.bo/. Accessed september 2018.

[41] Jors E, Konradsen F, Huici O, Morant RC, Volk J, Lander F (2016). Impact of training bolivian farmers on integrated pest management and diffusion of knowledge to neighboring farmers. J Agromedicine.

