# Correction: Pesticide exposure among Bolivian farmers: associations between worker protection and exposure biomarkers

**DOI:** 10.1038/s41370-021-00402-9

**Published:** 2021-12-15

**Authors:** Jessika Barrón Cuenca, Noemi Tirado, Max Vikström, Christian H. Lindh, Ulla Stenius, Karin Leander, Marika Berglund, Kristian Dreij

**Affiliations:** 1 Institute of Environmental Medicine, Karolinska Institutet, Box 210, SE-171 77 Stockholm, Sweden; 2 Genetic Institute, Medicine Faculty, Mayor of San Andres University, Saavedra Av., #2246 Miraflores, La Paz Bolivia; 3 Division of Occupational and Environmental Medicine, Lund University, 22363 Lund, Sweden

Correction to: *Journal of Exposure Science & Environmental Epidemiology* 10.1038/s41370-019-0128-3

The original article has been corrected.

The original version of this article was not published as Open Access article. The article was made open access retrospectively.

