# Supplementary material for: Pesticide exposure among Bolivian farmers: associations between worker protection and exposure biomarkers
Source: J Expo Sci Environ Epidemiol. 2019 Feb 20;30(4):730–42. doi: 10.1038/s41370-019-0128-3 (PMC8608618; doi:10.1038/s41370-019-0128-3)
Supplement: Supplementary file 1 — Supplementary Tables 1–4 [file 41370_2019_128_MOESM1_ESM.pdf]

SI Table 1. Variables and numerical values for calculating the PHI score.

Variable	Farmer answer	Points
Overall	Yes	3
	No	0
Hat	Yes	1
	No	0
Mask/Scarf	Yes	1
	No	0
Boots	Yes	1
	No	0
Gloves	Yes	1
	No	0
Glasses	Yes	1
	No	0
Apron	Yes	1
	No	0
Pesticide information source	Read labeled information/Agricultural engineer	1
	Own experience/Pesticide seller	0
Amount of pesticide used in each application	Recommended amount	1
	Doesn't measure at all	0
Chew coca meanwhile spraying	Yes	0
	No	1
Change spraying clothes	Yes	1
	No	0
Storage spraying clothes	Outside the house separately	1
	With all other clothes inside home	0
Wash clothes for spraying	Separately	1
	With all other clothes	0
Storage pesticides and equipment	Outside the house	1
	Inside the house	0

[1]A PHI score was calculated by summing points based on type of clothes used as PPE and handling and behavior during spraying pesticides. 16 points represented maximum protection and best behavior.

SI Table 2. Crops cultivated in the three different communities.

**Total[1]**		**Com1**		**Com2**		**Com3**	
Coca	68%	Tomatoes	71%	Coca	92%	Coca	89%
Banana	47%	Celery	66%	Banana	65%	Banana	58%
Tomatoes	39%	Corn	62%	Yucca	59%	Orange	49%
Yucca	39%	Fig	61%	Orange	50%	Yucca	43%
Orange	37%	Lettuce	49%	Rice	49%	Tangerine	36%
Corn	34%	Peach	47%	Pineapple	39%	Rice	32%
Rice	31%	Chard	45%	Tangerine	28%	Lemon	20%
Tangerine	24%	Beans	43%	Tomatoes	23%	Lima	19%
Celery	22%	Beetroot	38%	Corn	22%	Tomatoes	18%
Fig	20%	Prickly pear	38%	Green pods	17%	Corn	15%
Lettuce	19%	Cabbage	37%	Watermelon	15%	Cucumber	14%
Pineapple	16%	Parsley	33%	Avocado	14%	Peppers	12%
Cucumber	16%	Spinach	30%	Cucumber	13%	Green pods	12%
Peach	15%	Grapes	28%	Papaya	10%	Papaya	9%
Green bean	15%	Pear	28%	Carrots	10%	Passion fruit	8%
Cabbage	15%	Apple	26%	Peanuts	7%	Palmetto	6%
Chard	15%	Onion	19%	Onion	7%	Cabbage	6%
Beetroot	13%	Ice cream bean	19%	Walusa	6%	Avocado	5%
Prickly pear	12%	Cucumber	17%	Achojcha	6%	Walusa	4%
Parsley	12%	Sweet potato	17%	Lettuce	6%	Peanuts	3%
Green pods	12%	Peppers	12%	Lime	5%	Cabbage	3%
Apple	10%	Potatoes	11%	Mango	5%	Pineapple	2%
Peppers	10%	Lugma	9%	Apple	4%	Sugar cane	1%
Spinach	10%	Cantaloupe	8%	Ice cream bean	4%	Custard apple	1%
Pear	9%	Carrots	8%	Peppers	4%	Eggplant	1%
Lemon	9%	Broccoli	7%	Beans	4%	Carrots	1%
Grapes	9%	Green beans	7%	Lemon	3%	Lettuce	1%
Onion	9%	Plum	4%	Broad beans	3%	Parsley	1%
Lime	9%	Turnip	4%	Custard apple	2%	-	-
Ice cream bean	8%	Pumpkin	4%	Parsley	2%	-	-

[1]Data shows the first 35 crops of over 100 named

SI Table 3. General gynecological information and spraying behavior for women participating in the study.

	**Type of work**		**Total**	**Com1**	**Com2**	**Com3**
Miscarriages[1]	Total	No	52%	53%	55%	46%
		Yes	48%	47%	45%	54%
	Farmer	No	58%	54%	56%	39%
		Yes	42%	46%	44%	61%
	Not Farmer	No	57%	50%	50%	57%
		Yes	43%	50%	50%	43%
Malformed babies and still births[1]	Total	No	83%	90%	83%	78%
		Yes	17%	10%	17%	22%
	Farmer	No	85%	89%	81%	78%
		Yes	15%	11%	19%	22%
	Not Farmer	No	86%	0%	0%	79%
		Yes	14%	0%	0%	21%
Sprayed while pregnant[2]	Farmer	No	64%	57%	44%	70%
		Yes	36%	43%	56%	30%
Sprayed while breast feeding[2]	Farmer	No	69%	71%	56%	70%
		Yes	31%	29%	44%	30%

[1] Information over the 114 women who had been pregnant.
[2] Information over 94 women farmers.

SI Table 4. List of the 49 pesticides reported to be used by the study population and their frequency in total and by community.

PESTICIDE	Total	Com1	Com2	Com3
2,4 D	4%	0%	1%	13%
Abamectin B1a, B1b	9%	16%	11%	1%
Atrazine	0.4%	1%	0%	0%
Azoxystrobin (strobin) + Cyproconazole (Azoles)	4%	5%	2%	5%
Carbendazim	6%	0%	14%	3%
Carbosulfan	7%	0%	12%	8%
Carboxin (carboxiamide) + Thiram (dithiocarbamate)	2%	0%	0%	6%
Chlorfenapyr	5%	15%	0%	1%
Chlorothalonil (Tetrachloroisophthalonitrile)	2%	0%	0%	6%
Chlorpyrifos	13%	27%	6%	7%
Clethodim	2%	0%	3%	3%
Copper Sulphate	0.7%	2%	0%	0%
Cymoxanil (Unclassified)+ Mancozeb (Dithiocarbamate-ETU)	5%	3%	9%	2%
Cypermethrin	16%	26%	17%	6%
Difenoconazole (azole) + Propiconazole (azole)	6%	17%	0%	1%
Emamectin benzoate	0.4%	0%	0%	1%
Endosulfan	0.4%	0%	0%	1%
Etoxazole	0.4%	1%	0%	0%
Fenitrothion	0.7%	1%	1%	0%
Fenvalerate (pyrethroid) + Phoxim (Organophosphorus)	0.4%	0%	1%	0%
Fipronil	3%	10%	0%	0%
Flubendiamide	4%	13%	0%	0%
Fomesafen	1%	0%	1%	3%
Glufosinate-ammonium	0.7%	0%	0%	2%
Glyphosate	43%	0%	57%	67%
Imazaquin	0.4%	0%	0%	1%
Imidacloprid (Neonicotenoid) - Methomyl (N-Methyl Carbamate)	14%	1%	18%	21%
Lambdacyhalothrin	10%	9%	11%	11%
Lufenuron	0.4%	1%	0%	0%
Mancozeb	14%	16%	15%	10%
Methamidophos	65%	28%	91%	70%
Methomyl	12%	1%	11%	24%
Monocrotophos	0.4%	0%	0%	1%
Myclobutanil	1%	5%	0%	0%
Paraquat	52%	0%	71%	81%

Picloram + 2,4 D	10%	1%	4%	26%
Profenofos	10%	30%	2%	0%
Propanil	0.4%	0%	1%	0%
Propiconazole	5%	9%	0%	6%
Propineb	2%	3%	0%	2%
Quizalofop-P-ethyl	0.7%	2%	0%	0%
Spinosad	1%	5%	0%	0%
Sulfur	9%	30%	0%	0%
Tebuconazole	3%	7%	0%	3%
Thiodicarb	0.4%	1%	0%	0%
Thiophanate methyl	2%	2%	3%	0%
Triclorfon	1%	0%	4%	0%
Trifloxistrobin (Strobin) + Tebuconazole (triazole)	0.4%	1%	0%	0%
Triisopropanolamine salt of 4-amino-3,5,6-trichloro-picolinic acid	0.7%	0%	1%	1%

ENCUESTA DE EXPOSICIÓN A PLAGUICIDAS
PROYECTO ASDI SAREC

Nombre: …………………………………………………………………………………… Código: …………………..

Edad: …………………… Varón: ▢ Mujer: ▢ Nacido en: …………………………………………..

PARTE I.

1. Nivel educativo:
   Sin escolaridad ▢ Primaria ▢
   Secundaria ▢ Universidad/ Técnico ▢
2. Actividad laboral:
   Agricultor ▢ Estudiante ▢ Chofer ▢
   Profesor ▢ Empleado público ▢
   Otra: ………………………………………………………..
3. Usted consume verduras y frutas del lugar?
   Si / No (Respuesta No) Dónde compra los vegetales/frutas para su consumo?
   ……………………………………………………………………
4. Qué clase de vegetales y frutas crecen en esta región?………………………………………………………
   ……………………………………………………………………
   ……………………………………………………………………
   ……………………………………………………………………
5. Cuántas veces al día come vegetales? …………
6. Cuántas veces al día consume fruta? ……………
7. De donde proviene el agua que consume?
   Pozo ▢ Río ▢ Agua municipal ▢
   Otra: ………………………………………………………..
8. Alguna vez sintió un sabor diferente al comer sus vegetales? Si / No
9. Qué comió durante las últimas 24 horas?
   ……………………………………………………………………
   ……………………………………………………………………
   ……………………………………………………………………
   ……………………………………………………………………
10. Alguna vez sintió un olor desagradable alrededor de su casa cuando es tiempo de rociado de plaguicidas? Si / No
11. Usted tiene algún familiar agricultor(a)?
    Si / No
    Quién? ……………………………………
12. Alguna vez roció plaguicidas? Si / No
13. Usted ayuda a rociar plaguicidas? Si / No

PARTE II. SOLAMENTE AGRICULTORES.

14. Otra actividad aparte de ser agricultor?
    Estudiante ▢ Chofer ▢ Profesor ▢
    Empleado público ▢ Otra: ……………………..
15. Cuánto tiempo trabaja en agricultura?
    <1 año ▢ 1 – 3 años ▢
    4 – 7 años ▢ >/= 8 años ▢
16. Usted trabaja como agricultor todo el año o es trabajador solo en ciertas temporadas?
    Todo el año ▢ Sesiones ▢
17. Qué clase de cultivo(s) usted tiene?
    ……………………………………………………………………
    ……………………………………………………………………
    ……………………………………………………………………
18. Usted utilizó plaguicidas durante los últimos tres años? Si / No
19. Qué cultivos usted rocía con plaguicidas?
    ……………………………………………………………………
    ……………………………………………………………………
    ……………………………………………………………………
20. Quién fumiga los cultivos?
    Encuestado ▢ Otro trabajador ▢
    Pariente ▢
21. Quién le ayuda a fumigar?
    Padre ▢ Madre ▢ Esposa ▢
    Esposo ▢ Hermano(a) ▢ Hijo(a) ▢
22. En promedio cuántas horas al día usted trabaja en su huerta?
    <2hrs ▢ 2 – 4hrs ▢ >/= 5hrs ▢
23. Cuántos días al mes usted rocia plaguicidas?
    1 day ▢ 2-10 days ▢ 11–20 days ▢
    >20 days ▢
24. Qué plaguicida usted usa más frecuentemente?
    ……………………………………………………………………
    ……………………………………………………………………
    ……………………………………………………………………

25. Qué plaguicidas mezcla con mayor frecuencia? ……………………………………………… …………………………………………………………… …………………………………………………………… ……………………………………………………………
26. Cuánto plaguicida usted aplica en promedio?
    Cantidad recomendada ▢ No calcula ▢
27. Usted usa algún equipo de protección o ropa para rociar los plaguicidas?
    NS/NR ▢ No ▢ Sombrero ▢ Máscara ▢
    Overol ▢ Sandalias ▢ Botas ▢
    Pañoleta ▢ Guantes de goma ▢
    Lentes ▢ Mandil ▢
28. Usted se cambia sus ropas después de rociar? Si / No
29. Dónde guarda sus ropas para rociar?
    Con el resto de la ropa ▢ Patio ▢
    Con los animales ▢ En un lugar exclusivo ▢
30. Usted lava su ropa para rociar con el resto de la ropa de la familia? Si / No
31. De dónde usted obtiene información de cuánto plaguicida rociar?
    Recomen del envase ▢ Un supervisor ▢
    Experiencia ▢ Otro: ……………………………
32. Dónde guarda los plaguicidas y el equipo para rociar?
    Dentro la casa ▢ Patio ▢
    Con los animales ▢ En un lugar exclusivo ▢
33. Cómo elimina los envases de los plaguicidas?
    Quema ▢ Vota a la Basura ▢ Guarda ▢
    Otro: ……………………………………………………
34. De dónde proviene el agua para rociar su huerta? Agua municipal ▢
    Agua de pozo ▢ Río ▢
35. Alguna vez se sintió enfermo después de rociar plaguicidas? Si / No
    (Respuesta Si) Qué sintió?
    Ardor en ojos ▢ Calambres ▢
    Dolor de cabeza ▢ Mareos ▢
    Náuseas ▢ Vómitos ▢ Fiebre ▢
    Escalofríos ▢ Fatiga ▢
    Dificultad respiratoria ▢ Sudoración ▢
    Tos ▢ Dolor abdominal ▢
    Ojos rojos ▢ Lagrimeo ▢
    Visión borrosa ▢ Escozor ▢
    Enrojecimiento de la piel y ardor ▢
36. Usted mastica hojas de coca mientras rocía plaguicidas? Si / No

PARTE III. EVALUACION MÉDICA

HISTORIA DE LA ENFERMEDAD ACTUAL:
………………………………………………………………………
………………………………………………………………………
………………………………………………………………………

ANTECEDENTES PATOLÓGICOS FAMILIARES:
………………………………………………………………………
………………………………………………………………………

Padres agricultores: Si / No
Abuelos agricultores: Si / No
Historia familiar de Cáncer: Si / No
Quién? …………………………………………………
Historia familiar de Diabetes: Si / No
Quién? …………………………………………………
Consumo de medicamentos cotidianamente:
Si /No Cuáles?/ frecuencia……………………………
Fuma: Si / No Cuántos al día: …………………
Al mes: …………………
Bebe: Si / No Cuántos al día: …………
Al mes: ………. Qué bebida: ……………………

EXAMEN FÍSICO GENERAL:
Peso: ………..Kg Talla: ………..m IMC (peso/talla^2) : …………………
P.Arterial: …………mmHg FC: …………….lat/min
FR: …………….ciclos/min Glucosa (rapid test):………
………………………………………………………………………
………………………………………………………………………
………………………………………………………………………

Solo Mujeres:
Gestas: …… Partos: …….. Cesáreas: …….
Pérdidas: ……
Alguna vez roció estando embarazada: Si / No
Algún bebe malformado/muerto: Si / No
Dio de lactar a su bebe después de rociar:
Si / No

PESTICIDE EXPOSURE SURVEY
SIDA PROJECT

Name: …………………………………………………………………………………………. CODE: ……………….…………

Age: …………….. Man: ▢ Woman: ▢ Natural from: ………………………………………………….……..

PART I.

1. Educational attainment:
   No schooling ▢ Elementary School ▢ High school ▢ University/Technician ▢
2. Principal activity:
   Farmer ▢ Student ▢ Driver ▢ Teacher ▢ Public employee ▢
   Other: …………………….……..
3. Do you eat vegetables and fruits from the local area? Yes or No
   (If answer No) Where do you buy the vegetables for your consumption? …………………………………….……….
4. What kind of vegetables/fruits grow up in this region? ……………………………………………………………………..
   …….………
5. How often do you eat vegetables per day? ……………………………………………………………………………..………
6. How often do you eat fruits per day? …………………………………………………………………………………..………
7. Where does your drinking water come from? Well ▢ River ▢ Public Water Service ▢
   Other: …………………………………………………..
8. Have you ever tasted a different flavour in your vegetables? Yes or No
9. What did you eat during the last 24h? …………………………………………………………………………………………
   ………
10. Do you smell a disgusting odour around your house when it's time to spray pesticides? Yes or No
11. Do you have family in the agricultural activity? Yes or No Who? ……………………………………………
12. Have you ever sprayed with pesticides? Yes or No
13. Do you help to spray pesticides? Yes or No

PART II. FARMERS ONLY

14. Another activity besides farmer: Student ▢ Driver ▢ Teacher ▢ Public employee ▢ Other: ………..
15. Time of work on agriculture: <1 year ▢ 1 – 3 years ▢ 4 – 7 years ▢ >/= 8 years ▢
16. Do you work in the agricultural activity all the year or only you are a seasonal worker?
    All year ▢ Seasonal Worker ▢
17. Which kind of crops do you have? ……………………………………………………………………………………….……..
18. Did you use pesticides on your farm during the last three years? Yes or No
19. Which crop do you spray with pesticides? ……………………………………………………………………………………
    ………
20. Who treated the plants/crops with pesticides?
    Him/Herself ▢ Other farm worker ▢ Another relative ▢
21. Who helps you to spread the pesticide?
    Father ▢ Mother ▢ Wife ▢ Husband ▢ Brother/Sister ▢ Son/Daughter ▢
22. In average: How many hours per day do you work on your crop?
    < 2hrs ▢ 2 – 4hrs ▢ >/= 5hrs ▢
23. How many days per month did you spray pesticides?
    1 day ▢ 2 -10 days ▢ 11 – 20 days ▢ >20 days ▢
24. Which pesticide(s) do you use most frequently? (brand or commercial name) …………………………………
    ………
25. Which pesticides do you mix frequently? …………………………………………………………………………………
    ………

26. How much pesticides do you apply on the average? Recommended amount ▢ Doesn't measure ▢
27. Do you use any protective equipment or clothing when mixing pesticides?
    No ▢ Hat ▢ Mask ▢ Overall ▢ Sandals ▢ Boots ▢ Chinstrap ▢ Rubber gloves ▢ Glasses ▢ Plastic apron ▢
28. Did you change your clothes after spraying? Yes or No
29. Where do you save your clothes for spraying? With the rest of the clothes ▢ Outside the house ▢ With the animals ▢ In an exclusive place ▢
30. Do you wash your clothes with the rest of the family's clothes? Yes or No
31. Where do you find information about how to use pesticides? Bottle instructions ▢ Agricultural Engineer ▢ Own experience ▢ Another: ……………………………………………………………………….
32. Where do you store the pesticides and the fumigation equipment?
    Inside the house ▢ Outside the house ▢ With the animals ▢ Exclusive place ▢
33. How do you discard the empty bottles of pesticides?
    Burning ▢ Garbage ▢ Keep/store them ▢ Another: ……………………………………….
34. What is the primary source of irrigation water for your farm?
    Municipality water supply ▢ Well ▢ From the river ▢
35. Have you ever felt sick after sprayed pesticides? Yes or No (If is Yes) What did you feel?
    Burning eyes ▢ Cramps ▢ Headache ▢ Dizziness ▢ Vomiting ▢ Breathing difficulty ▢ Chili ▢ Fever ▢ Nausea ▢ Fainting ▢ Itching ▢ Sweating ▢ Cough ▢ Abdominal pain ▢ Red eyes ▢ Burning and redness of the skin ▢ Tearing ▢ Skin ulcers ▢ Blurred vision ▢
36. Do you chew coca leaves meanwhile you are spraying pesticides? Yes or No

PART III. MEDICAL EVALUATION

Actual illness history: ………
Familiar illness history: ……
Parents are/were farmers: Yes or No Grandparents were farmers: Yes or No
Familiar Cancer history: Yes or No Who?...
Familiar Diabetes history: Yes or No Who?...
Medicines consumption: Yes or No Witch medicines: …………………………………………………………….
Do you smoke? Yes or No # cigarettes per day: …………………… Per month: ………………
Do you drink alcohol? Yes or No # cups per day: ……………. Bottles per month: ………………..
Name of the drink: ………………………………………………………………………….

PHYSICAL EXAMINATION:

Weight: ……………..kg Height: …………….m Body Mass Index (BMI): …………….% CF: ……… lat/min
Blood pressure: …………… mmHg RF: ………. cycles/min Rapid glucose test: …………….

ONLY WOMEN

Number of: Gestations: ………. Births: ………… Caesarean: ………… Miss births: …………
Baby with malformation/stillbirth: …………………….
Have you ever sprayed when you were pregnant? Yes or No
Did you breastfeed your baby after spraying? Yes or No
